# Family therapy and EMDR after child abuse and neglect: moderating effects of child attachment style and PTSD symptoms on treatment outcome

**DOI:** 10.1080/20008066.2024.2416288

**Published:** 2024-10-25

**Authors:** Mara L. van der Hoeven, Samantha Bouwmeester, Nathalie E. F. Schlattmann, Ramón J.L. Lindauer, Irma M. Hein

**Affiliations:** a Department of Child and Adolescent Psychiatry, Amsterdam UMC, University of Amsterdam, Amsterdam, the Netherlands; b Out of the Box Plot & Tilburg University, Tilburg, the Netherlands; c Academic Center for Child and Adolescent Psychiatry, Levvel, Amsterdam, the Netherlands

**Keywords:** Child abuse & neglect, trauma treatment, family therapy, EMDR, posttraumatic stress symptoms, attachment style, moderator analyses, single-case experimental design, Abuso y negligencia infantil, tratamiento del trauma, terapia familiar, EMDR, síntomas de estrés postraumático, estilo de apego, análisis moderador, diseño experimental de caso único

## Abstract

**Background:** Effective and appropriate care and treatment for children in order to decrease the psychosocial problems that arose after experiencing child abuse and neglect (CAN) is of vital importance, given the severity of symptomatology that may develop.

**Objective:** The purpose of the present study was to examine whether attachment style and core cluster Posttraumatic Stress Disorder symptoms acted as moderators for treatment outcomes of a new integrative treatment model for trauma and attachment. In this treatment model, family therapy is combined with EMDR and obstacles for trauma processing are tackled first.

**Method:** we included children, ages 6–12 years, with a history of CAN, who did not respond to evidence-based trauma treatment. Target treatment outcomes were problems in attachment, posttraumatic stress symptoms, behaviour, and emotion regulation. We conducted a multiple-baseline ABC Single-Case Experimental Design (SCED). We categorized 12 participants into four groups of attachment style and core cluster PTSD symptoms: (1) non-disorganized & re-experiencing; (2) non-disorganized & avoidance/hyperarousal; (3) disorganized & re-experiencing; & (4) disorganized & avoidance/hyperarousal. We compared the four groups with each other and across time, and the interaction between groups and effect over time. We conducted non-parametric permutation tests and estimated *q*-values for false discovery rate control.

**Results:** Children with a disorganized attachment style had more severe symptomatology in general, except for posttraumatic stress symptoms. The treatment appeared more effective in targeting and successfully treating children with a non-disorganized attachment style, and specifically children with a non-disorganized attachment style and re-experiencing as core cluster PTSD symptoms.

**Conclusion:** Our study underlines the complexity of treating children who developed a complicated combination of symptomatology after CAN and calls for the continuous development of innovative interventions.

## HiGHLIGHTS

- The purpose of the present study was to identify possible moderators for the treatment outcome of children with early childhood abuse in the integrative treatment model for trauma and attachment.
- Type of attachment style and type of core cluster PTSD symptoms seem to moderate treatment outcomes.
- This study underlines the complexity of treating children who suffer from a complicated combination of posttraumatic stress symptoms, attachment problems, and behaviour and emotion regulation problems after child abuse & neglect.

## Abstract (es)

**Antecedentes:** La atención y el tratamiento eficaz y apropiado de los niños para disminuir los problemas psicosociales surgidos tras sufrir maltrato y abandono infantil (‘CAN’, por sus siglas en inglés) es de vital importancia, dada la gravedad de la sintomatología que pueden desarrollar.

**Objetivo:** El propósito del presente estudio fue examinar si el estilo de apego y los grupos centrales de síntomas del Trastorno de Estrés Postraumático actuaron como moderadores de los resultados del tratamiento de un nuevo modelo de tratamiento integrador para el trauma y el apego. En este modelo de tratamiento, la terapia familiar se combina con EMDR y se abordan en primer lugar los obstáculos para el procesamiento del trauma.

**Método:** Se incluyeron niños de 6 a 12 años con antecedentes de CAN que no respondieron al tratamiento del trauma basado en la evidencia. Los resultados del tratamiento escogido fueron problemas en el apego, síntomas de estrés postraumático, comportamiento y regulación de las emociones. Se llevó a cabo un Diseño Experimental de Caso Único (‘SCED’, por sus siglas en inglés) con múltiples líneas base ABC. Clasificamos a 12 participantes en cuatro grupos de estilo de apego y grupo de síntomas centrales de TEPT: (1) no desorganizado y reexperimentación; (2) no desorganizado y evitación/hiperactividad; (3) desorganizado y reexperimentación; y (4) desorganizado y evitación/hiperactividad. Se compararon los cuatro grupos entre sí y a lo largo del tiempo, así como la interacción entre los grupos y el efecto a lo largo del tiempo. Se realizaron pruebas de permutación no paramétricas y se estimaron los valores q para controlar la tasa de falsos descubrimientos.

**Resultados:** Los niños con un estilo de apego desorganizado presentaban una sintomatología más grave en general, excepto los síntomas de estrés postraumático. El tratamiento pareció ser más eficaz para tratar con éxito a los niños con un estilo de apego no desorganizado, y específicamente a los niños con un estilo de apego no desorganizado y reexperimentación como síntomas principales del TEPT.

**Conclusiones:** Nuestro estudio subraya la complejidad de tratar a niños que desarrollaron una combinación complicada de sintomatología después de una CAN y exige el desarrollo continuo de intervenciones innovadoras.

## 1. Introduction

Child abuse and neglect (CAN) in young children concerns early inter-relational traumatic events which may lead to a wide range of (developmental) problems in various areas, such as physical health problems, posttraumatic stress symptoms (PTSS), behavioural problems, attachment problems, anxiety problems, and depression (Carr et al., 2020). These problems may hamper future development with regard to social, educational, and societal functioning, and may threaten the continuation of the environment in which the child is brought up and, in case of foster care, the stability of placement (Konijn et al., 2019; Rostill-Brookes et al., 2011). Effective and appropriate care and treatment in order to decrease the psychosocial problems that arose after experiencing CAN is therefore of vital importance. Effective trauma-focused, attachment-focused, or family-oriented treatments have been developed for different types of problem behaviours. However, these treatments may be too narrow for this specific group of children due to the complex variety of problems that characterizes them. Extreme avoidance, behavioural and emotion dysregulation, and interpersonal reactivity in the child may lead to challenges that are hard to overcome in the treatment context (Struik et al., 2017; Wesselmann et al., 2017; Wesselmann & Shapiro, 2013).

For this vulnerable group of children, an integrated treatment model was developed, consisting of different techniques to better match their needs with regard to treatment (van der Hoeven et al., 2023). The Integrative Attachment Trauma Protocol for Children (IATP-C; Wesselmann et al., 2014) and its Dutch adaptation (Integratieve Gehechtheidsbevorderende Traumabehandeling voor Kinderen; IGT-K; Schlattmann et al., 2023) combines EMDR (Eye Movement Desensitization and Reprocessing; Shapiro, 2017) and family therapy. The treatment aims to diminish hurdles in order for trauma treatment to take place. Such barriers can be poor emotion tolerance, disruptive or avoidant child behaviours, and parent–child relationship difficulties. The original developers of IATP-C conducted case-series research and the results indicated decreases of attachment problems, behaviour problems, and traumatic stress symptoms (Wesselmann et al., 2018). Recently, the first experimental study on IGT-K was conducted. The results indicated that this integrative treatment model can be successful in decreasing problems for this population for whom limited treatment options are available due to difficulties of engaging them in evidence-based treatments and the lack of other available treatments (van der Hoeven et al., 2023).

Despite these promising results, questions remain about the influence of various child characteristics on treatment outcome within this population. As not all children showed an improvement on all outcome measures (van der Hoeven et al., 2023). Variation was found across the different cases and outcome measures. Two children showed clinical improvement on all four outcomes, two on three outcomes, three on one outcome (though this involved different outcomes per case), and one did not show clinical improvement on any outcome at all. This prompted further questions in terms of what mechanisms moderate treatment outcomes and for whom this treatment works best (Kraemer, 2016). It made us wonder whether pretreatment variables may also function as moderators in terms of treatment outcome. We developed a specific interest in the possible functioning of attachment style and PTSS as moderators, as these concepts can express themselves differently per individual.

In the vast literature on youth mental health care, we found little research on the contribution of attachment representations on differences in treatment outcome (Hornstra et al., 2022; Stefini et al., 2013). Attachment can be conceptualized as the child’s tendency to seek proximity and contact with an attachment figure (often a parent), especially in anxious and stressful situations (Ainsworth & Wittig, 1969; Bowlby, 1988). The concept involves the confidence of an individual in the availability of an attachment figure to function as a secure base and a safe haven. Three organized attachment styles have been identified (secure, anxious-avoidant, and anxious-ambivalent) and one disorganized style (Ainsworth et al., 1978; Main & Solomon, 1990). Securely attached children have confidence in the physical and emotional availability of their attachment figure. They form organized thoughts, behaviours, and emotions that enable them to adequately and accurately make their attachment needs known and expect a positive response from their caregiver (Schore, 2001). Insecure attachment indicates a lack of trust in the availability of the caregiver when the child is in need for consolation or protection. Anxious-avoidant and anxious-ambivalent patterns occur when caregivers are inconsistent emotionally available or responsive to the needs of the child. In order to maintain the relationship with the attachment figure, children might minimize their attachment needs as they have experienced consistent low sensitivity or rejection from their attachment figure (anxious-avoidant) or maximize their attachment needs as they have experienced inconsistent sensitivity from their attachment figure (anxious-ambivalent) (Ainsworth et al., 1978). Children who cannot be categorized in one of the three organized attachment categories are children who perceive their attachment figure as both a source of comfort and fear. This poses an unsolvable paradox and it prohibits the child to develop an organized strategy in relation to the use of help from the attachment figure. It leads to a combination of different types of insecure behaviours together with frightening reactions towards the attachment figure (Main & Hesse, 1990). Ambivalent and avoidant attachment styles are thought to be risk factors for maladjustment and the disorganized attachment style is thought to be most correlated with psychopathology (Main, 1996; Zeanah & Gleason, 2015). This blueprint of what can be expected in the interaction and relationship with another person can also influence interactions and relationships with others than the caregivers (Bowlby, 1980). Therefore, it is conceivable that attachment styles pass on into the therapeutic context and the way a child positions itself within therapy or interactions with the therapist. As the disorganized attachment style is thought to be most correlated with psychopathology and most robust to treat (de Wolff & Wildeman, 2020), it can be hypothesized that children with the highest scores on the disorganized style may show less improvement on the outcome measures in comparison with children with scores highest on the non-disorganized attachment style (ambivalent or avoidant).

Furthermore, we found little research available on the moderating role of PTSS-related variables on treatment outcome in youth. Studies showed that children with more severe PTSS at baseline achieved a smaller response in direct trauma-focused treatments (de Roos et al., 2021; Lindebø Knutsen et al., 2020; Wamser-Nanney et al., 2016). The potential moderating role of dysfunctional posttraumatic cognitions on treatment outcome was examined, with studies finding no evidence for such a moderating role (de Roos et al., 2021; Lindebø Knutsen et al., 2020). Trauma type as a moderator was also investigated, with different results. Some studies found no evidence (Danzi & La Greca, 2021; Goldbeck et al., 2016), whereas others found that effect sizes for treatment outcomes in terms of PTSS, internalizing problems, and functioning were larger for children who experienced sexual abuse (Kane et al., 2016; Silverman et al., 2008). Children with higher levels of emotion dysregulation showed less decrease in symptoms during treatment (Sharma-Patel & Brown, 2016). Children enrolled in IGT-K are characterized by relatively high levels of dysregulation and/or avoidance. These symptoms may complicate the course of treatment (Struik et al., 2017; Wesselmann et al., 2017; Wesselmann & Shapiro, 2013). Accordingly, it is imaginable that children with the highest scores on the avoidance and/or hyperarousal clusters show less improvement on the outcome measures in comparison with children with the highest scores on the re-experiencing cluster.

Moreover, it is possible that different combinations of type of attachment style and type of core cluster PTSD together result in different treatment outcomes across the population. As disorganized attachment style and avoidance or hyperarousal as core cluster PTSD symptoms are found to complicate the course of treatment, it is hypothesized that children with this combination of symptomatology show less improvement on all outcome measures compared to the other combinations. Children with a non-disorganized attachment style and re-experiencing as core cluster PTSD symptoms are subsequently hypothesized to show more improvement in comparison to the other combinations of attachment style and core cluster PTSD symptoms.

The purpose of the present study was to identify possible moderators for the treatment outcome of children in the integrative treatment model for trauma and attachment. We were particularly interested in whether attachment style (disorganized vs. non-disorganized) and core cluster PTSD symptoms (avoidance or hyperarousal vs. re-experiencing) acted as moderators for treatment outcomes with regard to levels of attachment problems, PTSS, behavioural problems, and emotion regulation problems.

## 2. Methods

### 2.1. Study design

This paper was part of a first study on the effectiveness of the integrative treatment model for trauma and attachment in children, which was described extensively elsewhere (van der Hoeven et al., 2023). The Medical Ethics Committee of the Amsterdam UMC, location AMC, approved this project under project number W17_169.

We conducted a multiple-baseline ABC Single-Case Experimental Design (SCED). Participants were randomized between baseline lengths of three, four, and five weeks (Kratochwill et al., 2010). The intervention consisted of 33 weeks and was divided into two phases: one phase with preparatory techniques and one phase of trauma processing interventions.

### 2.2. Setting and participants

We conducted the study at two academic centers for child and adolescent psychiatry in the Netherlands, where recruitment transpired between May 2018 and December 2020. Custodial guardians were asked for written informed consent and children for their oral informed consent. The following eligible criteria were set: (1) children aged between four and 12 years old in a stable living situation (either with biological, foster, adoptive, or other resource parents with whom it should be possible to build an attachment relationship) – if the parent participating in the treatment was responsible for the past abuse, it was required that the parent had undergone treatment and the abuse has stopped; (2) referred to specialized youth mental health care for assessment and treatment of PTSS, attachment problems, behaviour problems, and/or self-regulation problems and with clinical cut-off scores for at least three out of these four outcome measures; (3) a history of child maltreatment within their primary attachment relationships; and (4) a failed attempt of direct evidence-based trauma-focused treatments or a clinical judgment that the child was not able to participate in direct trauma-focused treatment, due to strong avoidant of dysregulated behaviours. The presence of Fetal Alcohol Syndrome, Autism Spectrum Disorder, a child IQ score of 70 or below, or a clinical judgment that current caregiver’s psychiatric problems would interfere with the (emotional) availability of the caregiver for the child during treatment were set as exclusion criteria.

Fifteen children were eligible for study participation. One participant withdrew consent shortly before the start of treatment due to unexpected changes in the care system of the child. One dropped out after six treatment sessions due to the caregivers not being able to make the time investment the treatment requires. One participant completed only seven measurements and therefore, could not be included in the analyses, although this participant did complete the treatment. Four participants completed the majority of measurements but dropped out of the study during the last treatment phase. Their reasons for dropping out of the study were not directly related to the treatment or their problems, as these concerned unexpected court rulings regarding placement or changes in visiting arrangements with the biological parents. Therefore, we decided to include these four participants and the eight participants who completed both the study and treatment in their entirety into the analyses of this current study. Of the 12 included participants within this study, seven were boys. The mean age of these 12 children at the moment of inclusion was 9.72 years (SD = 1.85; range = 6–12 years). We displayed participants’ background variables and pretreatment scores in Table 1.

**Table 1.** Participants’ background and pre-treatment variables.

Participant	Gender	Age (years)	Placement situation	Contact with biological parents	Length of placement (years; months)	Custodial guardian	Medication	AISI total^1^	CRIES total^1^	SDQ total^1^	BRIEF emotional control^1^	Baselinelength in weeks
004^2^	Male	9	Family home	No	2;6	State guardian	Yes	59*	23	26*	27*	5
005^3^	Male	8	Foster care within family	Yes	2;0	State guardian	No	54*	36*	21*	23	3
006^2^	Male	9	Foster care	Yes	2;0	State guardian	No	63*	51*	26*	25	4
007^2^	Female	10	Foster care	Yes	3;0	Biological parents	Yes	39	41*	28*	27*	4
009^2^	Male	9	Foster care	Yes	1;6	Biological parents	No	52*	35*	15*	16	5
010^2^	Male	6	Foster care	No	6;0	State guardian	Yes	68*	21	25*	29*	3
011^4^	Male	10	Biological mother	NA	10;0	Biological mother	No	73*	29	34*	30*	3
012^4^	Male	7	Biological mother	NA	7;4	Biological mother	Yes	35	30*	15*	26*	5
016^3^	Male	9	Foster care	Yes	2;6	State guardian	No	61*	23	20*	27*	3
017^2^	Female	10	Foster care	Yes	8;0	State guardian	No	85*	47*	23*	28*	5
018^2^	Male	10	Foster care within family	Yes	7;0	State guardian	No	52*	46*	21*	30*	3
020^2^	Female	8	Adoption	No	2;4	Adoptive parents	Yes	79*	33*	25*	28*	4
022^3^	Female	12	Foster care	Yes	1;5	Biological mother	No	62*	37*	16*	24	3
024^3^	Female	11	Foster care	No	7;4	State guardian	Yes	58*	45*	30*	30*	5

^1^ These four indices are the indices on which participants had to report clinical cut-off scores on at least three of these four indices. We indicated on which of these indices a participant scored a clinical cut-off score with a *.

^2^ These participants completed the treatment and the study in their entirety are therefore included in the analyses of this paper.

^3^ These participants completed the majority of measurements but dropped out of the study during the last teatment phase.

^4^ These participants dropped out of treatment shortly after the treatment onset or completed little measurements.

§One participant is not displayed in this table as consent was withdrawn shortly before the treatment onset.

### 2.3. Procedure

After consent was acquired, the child and his or her caregivers were assigned to a duo of therapists and the length of baseline was randomized. During the baseline and intervention phases, caregivers completed four questionnaires (CRIES, AISI, SDQ, and the emotion control scale of the BRIEF) on a weekly basis through telephone on a prefixed date.

### 2.4. Treatment and treatment fidelity

The treatment examined in the study was the ‘Integratieve Gehechtheidsbevorderende Traumabehandeling voor Kinderen’ (IGT-K), which was the Dutch adaptation of the Integrative Attachment Trauma Protocol for Children (Wesselmann et al., 2014; Wesselmann et al., 2018). The IGT-K combined family therapy with EMDR therapy. The protocol targeted obstacles that complicate the child’s utilization of trauma-focused treatments, such as extreme avoidance and dysregulated behaviours. The treatment started with a preparation phase of 17 weeks, in which the first sessions are with the caregivers alone for psycho-education. It carried on with ten caregiver-child sessions, in which work was done to strengthen attachment and enhancement of self-regulation (phase B). Consequently, it continued with an EMDR trauma processing phase of 16 weeks. For a detailed description of the treatment protocol, we refer to earlier publications and the manual for therapists (Schlattmann et al., 2023; van der Hoeven et al., 2023; Wesselmann et al., 2014; Wesselmann et al., 2018). A description of how the therapists were trained and how treatment fidelity was warranted can also be found there.

### 2.5. Measurements

In order to measure attachment problems, PTSS, emotion regulation difficulties, and behaviour problems, pre-and posttreatment as well as weekly measurements during the treatment were conducted. All questionnaires were filled in by the primary caregiver. We used to following measurements.

For attachment problems, we used the Attachment Insecurity Screening Inventory (AISI; Polderman & Kellaert-Knol, 2012). This questionnaire assesses primary caregivers’ perception of Insecure-Avoidant, Insecure-Ambivalent/Resistant, and Insecure-Disorganized attachment relationships with their child. The questionnaire holds 20 items with a 6-point Likert scale: never, sometimes, regularly, often, very often, and always. The three subscales together form a total score of insecure attachment that can range between 20–120. A score of 50 or higher indicates an insecure attachment. The Dutch version of AISI holds acceptable psychometric properties, with Cronbach’s α found between .65 and .80 (Spruit et al., 2018).

We used the caregiver-report version of the Child Revised Impact of Events Scale to measure PTSS (CRIES-13; Perrin et al., 2005; Verlinden et al., 2014), which consists out of 13 items. These items can be scored as never (0), rarely (1), sometimes (3), or often (5). The items can be clustered into three scales: avoidance, re-experiencing, and arousal. The total score can range between zero and 65. A score of 30 or higher indicates a higher risk for the development of PTSD. The Dutch version of the CRIES-13 has good psychometric properties, with Cronbach’s α of .87 (Verlinden et al., 2014).

We measured emotional and behavioural difficulties with the Strengths and Difficulties Questionnaire (SDQ; Goodman, 1997; van Widenfelt et al., 2003). This is a 25 item-questionnaire describing positive and negative attributions of children and adolescents. The questionnaire consists of 5 subscales: emotional symptoms, conduct symptoms, hyperactivity-inattention symptoms, peer problems, and prosocial behaviour. The first four scales together form a total difficulties score, with higher scores reflecting more difficulties. The total score can range between zero and 40. In our study, we used the total difficulties score. The Dutch version of the SDQ has good psychometric properties, with Cronbach’s α of .70 (Muris et al., 2003).

We used the subscale Emotional Control of the Behavior Ratings Inventory Executive Function to measure self-regulation problems (BRIEF; Gioia et al., 2000a; Huizinga & Smidts, 2009). The scores of this scale range between 10 and 30. Higher scores indicate more difficulties with emotional control. The BRIEF in total has shown good psychometric properties, with Cronbach’s α between .78 and .90 (Gioia et al., 2000b; Huizinga & Smidts, 2009, 2010).

### 2.6. Data analysis

We focused on potential moderating effects of attachment style (disorganized vs. non-disorganized) and core cluster PTSD symptoms (avoidance/hyperarousal vs. re-experiencing) on treatment response. Treatment outcome measures were levels of attachment problems, PTSS, behavioural problems, and emotion regulation problems. Regarding attachment scores, we examined which attachment style (anxious-avoidant, anxious-ambivalent, disorganized) had the relative highest score per participant on the AISI. Subsequently, we grouped the participants within the anxious-avoidant and anxious-ambivalent groups together into one group ‘non-disorganized. Regarding PTSD-symptoms, we examined which of the three clusters (re-experiencing, avoidance, hyperarousal) had the highest score per individual on the CRIES. Subsequently, we grouped the participants within the avoidance and the hyperarousal groups into one group ‘avoidance/hyperarousal’. We categorized participants into four groups for the attachment and the core cluster PTSD symptoms variable based on the participants’ subscale of the AISI or CRIES with relative highest scores measured pretreatment. The four groups were as follows: (1) non-disorganized & re-experiencing; (2) non-disorganized & avoidance/hyperarousal; (3) disorganized & re-experiencing; and (4) disorganized & avoidance/hyperarousal.

We calculated the effect sizes in Cohen’s *d* to assess the value of the intervention per outcome measure per group. We compared the four groups, the three phases (baseline, preparation, and treatment) and the interaction between the groups and effect over time. First, for each of the four groups, we aggregated all observations within one phase per outcome measure and calculated the mean. For the main effect of group, we evaluated whether the groups differed in means, aggregated over all observations in the three phases. For the main effect of time, we aggregated the observations of the different groups within a phase, and compared the means of the three phases. For the interaction effect, we evaluated whether the differences between the phases differed for the four groups.

#### 2.6.1. Randomization tests

With regard to the randomization tests, we followed Ernst (2004) and conducted non-parametric permutation testing through the use of a Shiny App Single Case Designs, which was running on *R* version 4.3.2 and, among other things, made use of the package ‘shiny’ (Bouwmeester, 2021). For the group comparisons, first, the observed mean differences were calculated by aggregating the scores within a group for each of the three phase comparisons. Next, the scores of the two phases that were compared were shuffled and then randomly assigned to one of the phases. This was done for each of the four groups and repeated 1000 times. Subsequently, we calculated the squared mean differences for these randomly formed phases and summed them. Then, the *p*-value was calculated as the proportion of randomly formed *t*-values was more extreme than the observed *t*-values. When the overall *p*-value was significant, we performed post-hoc analyses to check which of the four groups differed. We estimated *q*-values for false discovery rate control (Storey, 2002, 2003). Since many statistical tests are performed, *q* values will be reported in addition to the *p*-values. The q-value is an analog of the *p*-value that incorporates multiple testing correction. The *q*-value is defined as the minimum false discovery rate at which an observed score is deemed significant.

## 3. Results

**Figure 1.**
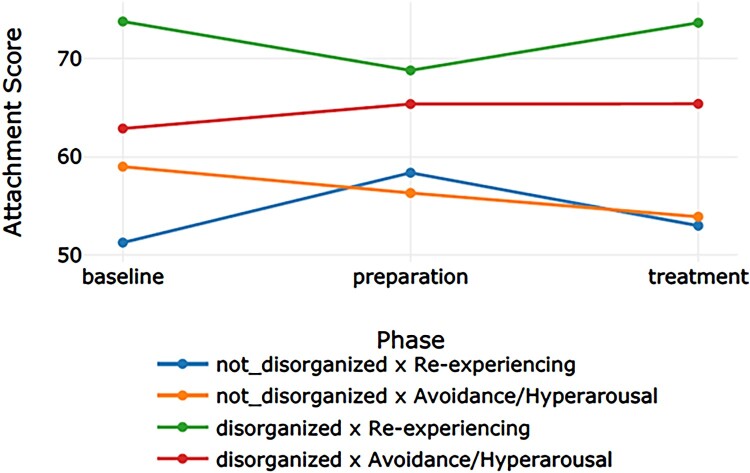
Graph corresponding with the moderator analyses on attachment problems.

**Figure 2.**
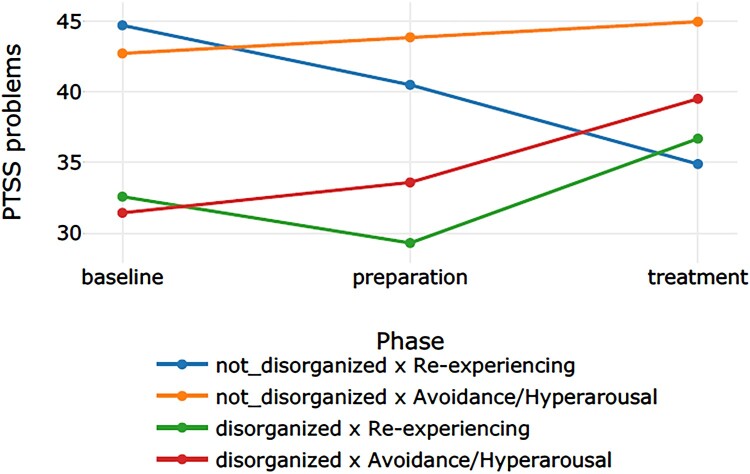
Graph corresponding with the moderator analyses on PTSS.

**Figure 3.**
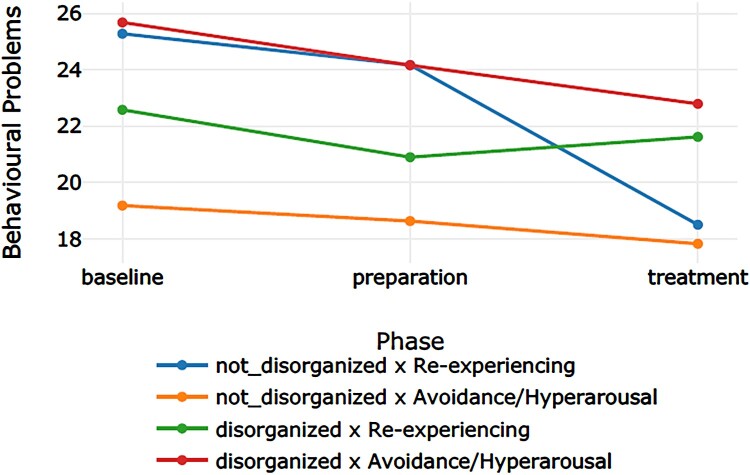
Graph corresponding with the moderator analyses on behavioural problems.

**Figure 4.**
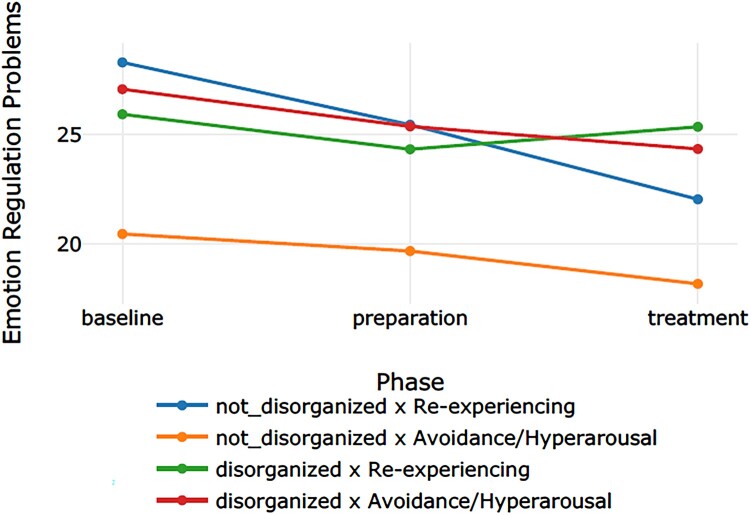
Graph corresponding with the moderator analyses on emotion regulation problems.

Table 2 displays the categorization of the 12 participants into the four groups. We refer to Table 3 for the descriptive values. Table 4 presents the effect sizes (Cohen’s *d*) for the phase comparisons per outcome measure for each of the four groups. Table 5 displays the *p* – and *q-*values for the main effect for group, the main effect for time (the three phases: baseline, preparation, and intervention), and the interaction effect (time × group) for the four outcome measures. Subsequently, Tables 6–8 display the results of the post-hoc tests for group, time, and time × group. Figures 1–4 display the course of symptoms of each of the four groups per outcome measure.

**Table 2.** Classification of participants into four groups according to the attachment category and the core cluster PTSD symptom category.

Type of core cluster PTSD symptoms	Type of attachment style		Total
	Disorganized	Non-disorganized	
Avoidance/hyperarousal	5	2	7
Re-experiencing	3	2	5
Total	8	4	12

**Table 3.** Descriptive values for the four groups on the four outcome measures.

Phase	Total attachment problems		Total PTSS		Behavioural problems		Emotion regulation problems	
	Mean	SD	Mean	SD	Mean	SD	Mean	SD
Non-Disorganized & Re-Experiencing								
Baseline	51.29	5.06	44.71	12.97	25.29	4.98	28.29	2.82
Preparation	58.38	7.74	40.50	12.86	24.18	5.44	25.44	3.9
Treatment	53.00	8.43	34.89	12.77	18.50	6.04	22.04	4.26
Non-Disorganized & Avoidance/Hyperarousal								
Baseline	59.00	13.32	42.73	9.06	19.18	3.78	20.45	1.98
Preparation	56.33	17,47	43.87	12.51	18.63	4.58	19.67	3.4
Treatment	53.91	14.45	44.97	10.64	17.82	5.75	18.18	3.24
Disorganized & Re-Experiencing								
Baseline	73.75	12.00	32.58	13.15	22.58	4.21	25.92	4.5
Preparation	68.78	11.15	29.30	9.27	20.90	4.75	24.32	3.99
Treatment	73.62	11.36	36.69	7.36	21.62	4.15	25.34	3.91
Disorganized & Avoidance/Hyperarousal								
Baseline	62.88	7.95	31.44	5.28	25.69	4.72	27.06	2.21
Preparation	65.36	13.38	33.59	7.17	24.17	5.64	25.36	4.82
Treatment	65.38	16.81	39.51	16.34	22.79	7.53	24.33	6.83

SD = standard deviation.

**Table 4.** Effect sizes (Cohen’s d) for the phase comparisons per outcome measure for each of the four groups.

Comparison	Attachment problems	PTSS	Behavioural problems	Emotion regulation problems
Non-Disorganized & Re-Experiencing				
Baseline vs. preparation	1.11	.33	.24	.85
Preparation vs. treatment	.67	.44	.99	.83
Baseline vs. treatment	.25	.76	1.36	1.77
Non-Disorganized & Avoidance/Hyperarousal				
Baseline vs. preparation	.17	.11	.13	.29
Preparation vs. treatment	.15	.10	.16	.45
Baseline vs. treatment	.37	.23	.29	.87
Disorganized & Re-Experiencing				
Baseline vs. preparation	.43	.29	.38	.38
Preparation vs. treatment	.43	.89	.16	.26
Baseline vs. treatment	.01	.40	.23	.14
Disorganized & Avoidance/Hyperarousal				
Baseline vs. preparation	.23	.35	.29	.48
Preparation vs. treatment	0	.50	.21	.18
Baseline vs. treatment	.20	.75	.47	.60

**Table 5.** *p*-values & *q*-values for main effect group, main effect time, and interaction effect time x group for the four outcome measures.

Outcome measure	Main effect group		Main effect time		Interaction effect (Time x Group)	
	*p*-value	*q*-value	*p*-value	*q*-value	*p*-value	*q*-value
Attachment Problems	<.001	<.001	.926	.997	<.001	<.001
PTSS	<.001	<.001	.458	.538	.001	<.001
Behavioural Problems	<.001	<.001	.011	.014	.002	.003
Emotion Regulation Problems	<.001	<.001	.001	<.001	<.001	<.001

**Table 6.** Post-hoc tests for groups for the four outcome measures.

Comparison	MD	*p*-value	*q*-value
Attachment problems			
Non-Disorganized & Re-Experiencing vs. Non-Disorganized & Avoidance/Hyperarousal	−.27	.910	.474
Non-Disorganized & Re-Experiencing vs. Disorganized & Re-Experiencing	−15.50	<.001	<.001
Non-Disorganized & Re-Experiencing vs. Disorganized & Avoidance/Hyperarousal	−9.56	<.001	<.001
Non-Disorganized & Avoidance/Hyperarousal vs. Disorganized & Re-Experiencing	−15.23	<.001	<.001
Non-Disorganized & Avoidance/Hyperarousal vs. Disorganized & Avoidance/Hyperarousal	−9.29	<.001	<.001
Disorganized & Re-Experiencing vs. Disorganized & Avoidance/Hyperarousal	5.94	<.001	<.001
PTSS			
Non-Disorganized & Re-Experiencing vs. Non-Disorganized & Avoidance/Hyperarousal	−5.49	.005	.004
Non-Disorganized & Re-Experiencing vs. Disorganized & Re-Experiencing	6.56	<.001	<.001
Non-Disorganized & Re-Experiencing vs. Disorganized & Avoidance/Hyperarousal	3.44	.074	.051
Non-Disorganized & Avoidance/Hyperarousal vs. Disorganized & Re-Experiencing	12.05	<.001	<.001
Non-Disorganized & Avoidance/Hyperarousal vs. Disorganized & Avoidance/Hyperarousal	9.93	<.001	<.001
Disorganized & Re-Experiencing vs. Disorganized & Avoidance/Hyperarousal	−3.13	.078	.051
Behavioural Problems			
Non-Disorganized & Re-Experiencing vs. Non-Disorganized & Avoidance/Hyperarousal	3.59	<.001	<.001
Non-Disorganized & Re-Experiencing vs. Disorganized & Re-Experiencing	.63	.514	.299
Non-Disorganized & Re-Experiencing vs. Disorganized & Avoidance/Hyperarousal	−1.94	.020	.015
Non-Disorganized & Avoidance/Hyperarousal vs. Disorganized & Re-Experiencing	−2.96	.001	<.001
Non-Disorganized & Avoidance/Hyperarousal vs. Disorganized & Avoidance/Hyperarousal	−5.53	<.001	<.001
Disorganized & Re-Experiencing vs. Disorganized & Avoidance/Hyperarousal	−2.57	.002	.002
Emotion Regulation Problems			
Non-Disorganized & Re-Experiencing vs. Non-Disorganized & Avoidance/Hyperarousal	5.14	<.001	<.001
Non-Disorganized & Re-Experiencing vs. Disorganized & Re-Experiencing	−.51	.538	.299
Non-Disorganized & Re-Experiencing vs. Disorganized & Avoidance/Hyperarousal	−.91	.223	.139
Non-Disorganized & Avoidance/Hyperarousal vs. Disorganized & Re-Experiencing	−5.65	<.001	<.001
Non-Disorganized & Avoidance/Hyperarousal vs. Disorganized & Avoidance/Hyperarousal	−6.05	<.001	<.001
Disorganized & Re-Experiencing vs. Disorganized & Avoidance/Hyperarousal	−.40	.550	.299

MD = Mean Difference.

**Table 7.** Post-hoc tests for time for two outcome measures.

Comparison	MD	*p-*value	*q*-value
Behavioural Problems			
Baseline vs. Preparation	1.23	.200	>.999
Baseline vs. Treatment	2.95	.005	.050
Treatment vs. Preparation	1.73	.004	.050
Emotion Regulation Problems			
Baseline vs. Preparation	1.59	.055	.330
Baseline vs. Treatment	2.92	<.001	.003
Treatment vs. Preparation	1.32	.011	.083

MD = Mean Difference.

**Table 8.** Post-hoc tests for the interaction effect (Time × Group) for the four outcome measures.

Comparison	MD	*p-*value	*q-*value
Attachment Problems – Baseline vs. Preparation			
Non-Disorganized & Re-Experiencing vs. Non-Disorganized & Avoidance/Hyperarousal	−9.77	.196	.618
Non-Disorganized & Re-Experiencing vs. Disorganized & Re-Experiencing	−12.07	.119	.500
Non-Disorganized & Re-Experiencing vs. Disorganized & Avoidance/Hyperarousal	−4.61	.337	.729
Non-Disorganized & Avoidance/Hyperarousal vs. Disorganized & Re-Experiencing	−2.30	.753	.969
Non-Disorganized & Avoidance/Hyperarousal & Disorganized & Avoidance/Hyperarousal	5.16	.522	.874
Disorganized & Re-Experiencing vs. Disorganized & Avoidance/Hyperarousal	7.46	.130	.518
Attachment Problems – Baseline vs. Treatment			
Non-Disorganized & Re-Experiencing vs. Non-Disorganized & Avoidance/Hyperarousal	−6.80	.356	.737
Non-Disorganized & Re-Experiencing vs. Disorganized & Re-Experiencing	−1.84	.798	.969
Non-Disorganized & Re-Experiencing vs. Disorganized & Avoidance/Hyperarousal	.80	.858	.969
Non-Disorganized & Avoidance/Hyperarousal vs. Disorganized & Re-Experiencing	4.96	.443	.798
Non-Disorganized & Avoidance/Hyperarousal & Disorganized & Avoidance/Hyperarousal	7.59	.268	.653
Disorganized & Re-Experiencing vs. Disorganized & Avoidance/Hyperarousal	2.64	.544	.874
Attachment Problems – Preparation vs. Treatment			
Non-Disorganized & Re-Experiencing vs. Non-Disorganized & Avoidance/Hyperarousal	2.97	.637	.965
Non-Disorganized & Re-Experiencing vs. Disorganized & Re-Experiencing	10.22	.089	.449
Non-Disorganized & Re-Experiencing vs. Disorganized & Avoidance/Hyperarousal	5.40	.196	.618
Non-Disorganized & Avoidance/Hyperarousal vs. Disorganized & Re-Experiencing	7.25	.260	.653
Non-Disorganized & Avoidance/Hyperarousal & Disorganized & Avoidance/Hyperarousal	2.43	.709	.969
Disorganized & Re-Experiencing vs. Disorganized & Avoidance/Hyperarousal	−4.82	.247	.653
PTSS – Baseline vs. Preparation			
Non-Disorganized & Re-Experiencing vs. Non-Disorganized & Avoidance/Hyperarousal	5.36	.424	.783
Non-Disorganized & Re-Experiencing vs. Disorganized & Re-Experiencing	.93	.909	.976
Non-Disorganized & Re-Experiencing vs. Disorganized & Avoidance/Hyperarousal	6.37	.147	.556
Non-Disorganized & Avoidance/Hyperarousal vs. Disorganized & Re-Experiencing	−4.43	.548	.874
Non-Disorganized & Avoidance/Hyperarousal & Disorganized & Avoidance/Hyperarousal	1.01	.883	.969
Disorganized & Re-Experiencing vs. Disorganized & Avoidance/Hyperarousal	5.43	.207	.627
PTSS – Baseline vs. Treatment			
Non-Disorganized & Re-Experiencing vs. Non-Disorganized & Avoidance/Hyperarousal	12.06	.074	.411
Non-Disorganized & Re-Experiencing vs. Disorganized & Re-Experiencing	13.93	.043	.362
Non-Disorganized & Re-Experiencing vs. Disorganized & Avoidance/Hyperarousal	17.90	<.001	.008
Non-Disorganized & Avoidance/Hyperarousal vs. Disorganized & Re-Experiencing	1.86	.772	.969
Non-Disorganized & Avoidance/Hyperarousal & Disorganized & Avoidance/Hyperarousal	5.83	.360	.737
Disorganized & Re-Experiencing vs. Disorganized & Avoidance/Hyperarousal	3.97	.321	.715
PTSS – Preparation vs. Treatment			
Non-Disorganized & Re-Experiencing vs. Non-Disorganized & Avoidance/Hyperarousal	6.71	.236	.653
Non-Disorganized & Re-Experiencing vs. Disorganized & Re-Experiencing	13.00	.029	.314
Non-Disorganized & Re-Experiencing vs. Disorganized & Avoidance/Hyperarousal	11.53	.001	.038
Non-Disorganized & Avoidance/Hyperarousal vs. Disorganized & Re-Experiencing	6.29	.276	.653
Non-Disorganized & Avoidance/Hyperarousal & Disorganized & Avoidance/Hyperarousal	4.82	.423	.783
Disorganized & Re-Experiencing vs. Disorganized & Avoidance/Hyperarousal	−1.47	.699	.969
Behaviour Problems – Baseline vs. Preparation			
Non-Disorganized & Re-Experiencing vs. Non-Disorganized & Avoidance/Hyperarousal	.56	.863	.969
Non-Disorganized & Re-Experiencing vs. Disorganized & Re-Experiencing	−.57	.848	.969
Non-Disorganized & Re-Experiencing vs. Disorganized & Avoidance/Hyperarousal	−.41	.841	.969
Non-Disorganized & Avoidance/Hyperarousal vs. Disorganized & Re-Experiencing	−1.13	.748	.969
Non-Disorganized & Avoidance/Hyperarousal & Disorganized & Avoidance/Hyperarousal	−.97	.766	.969
Disorganized & Re-Experiencing vs. Disorganized & Avoidance/Hyperarousal	.16	.915	.976
Behaviour Problems – Baseline vs. Treatment			
Non-Disorganized & Re-Experiencing vs. Non-Disorganized & Avoidance/Hyperarousal	5.43	.071	.411
Non-Disorganized & Re-Experiencing vs. Disorganized & Re-Experiencing	5.82	.076	.411
Non-Disorganized & Re-Experiencing vs. Disorganized & Avoidance/Hyperarousal	3.89	.027	.314
Non-Disorganized & Avoidance/Hyperarousal vs. Disorganized & Re-Experiencing	.40	.872	.969
Non-Disorganized & Avoidance/Hyperarousal & Disorganized & Avoidance/Hyperarousal	−1.53	.570	.881
Disorganized & Re-Experiencing vs. Disorganized & Avoidance/Hyperarousal	−1.93	.320	.715
Behaviour Problems – Preparation vs. Treatment			
Non-Disorganized & Re-Experiencing vs. Non-Disorganized & Avoidance/Hyperarousal	4.87	.052	.385
Non-Disorganized & Re-Experiencing vs. Disorganized & Re-Experiencing	6.40	.016	.303
Non-Disorganized & Re-Experiencing vs. Disorganized & Avoidance/Hyperarousal	4.30	.016	.303
Non-Disorganized & Avoidance/Hyperarousal vs. Disorganized & Re-Experiencing	1.53	.554	.874
Non-Disorganized & Avoidance/Hyperarousal & Disorganized & Avoidance/Hyperarousal	−.56	.856	.969
Disorganized & Re-Experiencing vs. Disorganized & Avoidance/Hyperarousal	−2.09	.247	.653
Emotion Regulation Problems – Baseline vs. Preparation			
Non-Disorganized & Re-Experiencing vs. Non-Disorganized & Avoidance/Hyperarousal	2.06	.424	.783
Non-Disorganized & Re-Experiencing vs. Disorganized & Re-Experiencing	1.24	.650	.965
Non-Disorganized & Re-Experiencing vs. Disorganized & Avoidance/Hyperarousal	1.14	.464	.798
Non-Disorganized & Avoidance/Hyperarousal vs. Disorganized & Re-Experiencing	−.82	.754	.969
Non-Disorganized & Avoidance/Hyperarousal & Disorganized & Avoidance/Hyperarousal	−.92	.741	.969
Disorganized & Re-Experiencing vs. Disorganized & Avoidance/Hyperarousal	−.10	.951	1.00
Emotion Regulation Problems – Baseline vs. Treatment			
Non-Disorganized & Re-Experiencing vs. Non-Disorganized & Avoidance/Hyperarousal	3.97	.116	.501
Non-Disorganized & Re-Experiencing vs. Disorganized & Re-Experiencing	5.68	.035	.331
Non-Disorganized & Re-Experiencing vs. Disorganized & Avoidance/Hyperarousal	3.52	.022	.314
Non-Disorganized & Avoidance/Hyperarousal vs. Disorganized & Re-Experiencing	1.71	.457	.798
Non-Disorganized & Avoidance/Hyperarousal & Disorganized & Avoidance/Hyperarousal	−.45	.862	.969
Disorganized & Re-Experiencing vs. Disorganized & Avoidance/Hyperarousal	−2.16	.188	.618
Emotion Regulation Problems – Preparation vs. Treatment			
Non-Disorganized & Re-Experiencing vs. Non-Disorganized & Avoidance/Hyperarousal	1.91	.400	.783
Non-Disorganized & Re-Experiencing vs. Disorganized & Re-Experiencing	4.43	.056	.385
Non-Disorganized & Re-Experiencing vs. Disorganized & Avoidance/Hyperarousal	2.38	.107	.501
Non-Disorganized & Avoidance/Hyperarousal vs. Disorganized & Re-Experiencing	2.52	.261	.653
Non-Disorganized & Avoidance/Hyperarousal & Disorganized & Avoidance/Hyperarousal	.47	.840	.969
Disorganized & Re-Experiencing vs. Disorganized & Avoidance/Hyperarousal	−2.05	.167	.602

MD = Mean Difference.

### 3.1. Attachment problems

A small effect size was found for the non-disorganized × avoidance/hyperarousal group. There was a main effect for group, *p *< .001. The post-hoc tests showed that, averaged over all phases, all groups differed from each other; except for the non-disorganized × re-experiencing group and the non-disorganized × avoidance/hyperarousal group, *p *= .910. The disorganized × re-experiencing group had the highest mean, followed by the disorganized × avoidance/hyperarousal group. The two groups of children with a non-disorganized attachment style had the lowest means. There was no significant effect for time, *p *= .926, indicating that the means for baseline, preparation, and treatment did not differ when all groups were put together. The results showed a significant interaction effect for time × group, *p *< .001. However, the post-hoc tests indicated that none of the pairwise comparisons had a *p*-value smaller than .05.

### 3.2. PTSS

A medium effect size was found for the non-disorganized x re-experiencing group. There was a main effect for group, *p *< .001. The post-hoc tests showed that, averaged over all phases, groups differed from each other, except for the non-disorganized x re-experiencing group and the disorganized × avoidance/hyperarousal group, *p *= .074, and the disorganized x re-experiencing group and the disorganized × avoidance/hyperarousal group, *p *= .078. The non-disorganized × avoidance/hyperarousal group had the highest mean, followed by the non-disorganized × re-experiencing group, the disorganized × avoidance/hyperarousal group, and the disorganized × re-experiencing group. There was no significant effect for time, *p *= .458, indicating that the means for baseline, preparation, and treatment did not differ when all groups were put together. The results showed a significant interaction effect for time × group, *p *= .001. The post-hoc tests indicated four significant comparisons. The decrease in scores from the baseline to the treatment phase in the non-disorganized × re-experiencing group differed significantly from the increase in scores in the disorganized × re-experiencing group, *p *= .043, and the disorganized × avoidance/hyperarousal group, *p *< .001. The same result was found for the difference between the preparation and the treatment phase: the decrease in scores in the non-disorganized × re-experiencing group differed significantly from the increase in scores in the disorganized × re-experiencing group, *p *= .029 and the disorganized × avoidance/hyperarousal group, *p *= .001.

### 3.3. Behaviour problems

A large effect size was found for the non-disorganized × re-experiencing group. There was a main effect for group, *p *< .001. The post-hoc tests showed that, averaged over all phases, all groups differed from each other except for the non-disorganized × re-experiencing group and the disorganized × re-experiencing group, *p *= .514. The disorganized × avoidance/hyperarousal group had the highest mean, followed by the non-disorganized × re-experiencing group and the disorganized × re-experiencing group. The non-disorganized × avoidance/hyperarousal group had the lowest mean. There was a significant effect for time, *p *= .011. Post-hoc test showed that scores in the baseline were higher than scores in the treatment, *p *= .005, and scores in the preparation were higher than scores in the treatment, *p *= .004. The results showed a significant interaction effect for time × group, *p *= .002. The post-hoc tests indicated three significant comparisons. The decrease in scores from the baseline to the treatment in the non-disorganized × re-experiencing group was larger than the decrease in scores in the disorganized × avoidance/hyperarousal group, *p *= .027. The same result was found for the difference between the preparation and the treatment phase: the decrease in scores in the non-disorganized × re-experiencing group was larger than the decrease in scores in the disorganized × avoidance/hyperarousal group, *p *= .016. There was also a significant difference in the decrease in scores between the non-disorganized × re-experiencing group and the disorganized × re-experiencing group from the preparation to the treatment phase, *p *= .016.

### 3.4. Emotion regulation problems

A large effect size was found for both non-disorganized groups and a medium for the disorganized × avoidance/hyperarousal group. There was a main effect for group, *p *< .001. The post-hoc tests showed that, averaged over all phases, the mean of the non-disorganized x avoidance/hyperarousal group was lower than the means of the three other groups, which did not differ from each other. There was a significant effect for time, *p *= .001. Post-hoc test showed that scores in the baseline were higher than scores in the treatment, *p *= <.001, and scores in the preparation were higher than scores in the treatment, *p *= .011. Although for the latter, the *q-*value turned out non-significant (*q *= .083). The results showed a significant interaction effect for time × group, *p *< .001. The post-hoc tests indicated two significant comparisons. The decrease in scores from the baseline to the treatment in the non-disorganized × re-experiencing group was larger than the decrease in scores in the disorganized × avoidance/hyperarousal group, *p *= .022 (although the *q*-value turned out non-significant: *q *= .314), and disorganized × re-experiencing group, *p *= .035.

## 4. Discussion

In this study, we aimed to identify possible moderators for treatment response in children participating in a new integrative treatment model for trauma and attachment. We examined attachment style (disorganized vs. non-disorganized) and core cluster PTSD symptoms (avoidance or hyperarousal vs. re-experiencing) as moderators for treatment outcomes with regard to levels of attachment problems, PTSS, behavioural problems, and emotion regulation problems.

In general, it seemed that children with a disorganized attachment style experienced more severe levels of symptoms overall. These findings are in line with previous research indicating that children with a disorganized attachment style have higher levels of externalizing problem behaviour than children with the other insecure styles (Badovinac et al., 2021; Fearon et al., 2010; Groh et al., 2017; O'Connor et al., 2011). Disorganized attachment is thought to be linked to emotion regulation processes: children who experience their caregiver both as a source of safety and fear, may perceive the world as unpredictable and frightening and they may make less use of problem-solving coping, as they have missed opportunities to develop appropriate emotion regulation strategies (Brumariu, 2015; Brumariu et al., 2012; Lyons-Ruth & Jacobvitz, 2008). These vulnerabilities in terms of attachment and emotion regulation may lead to more behavioural or emotional control difficulties, as disorganized attachment is found to be associated with both internalizing as externalizing behaviours (Walsh et al., 2008). Children with a non-disorganized attachment style and re-experiencing had more behaviour problems and emotion regulation problems compared to children with a non-disorganized attachment style and avoidance/hyperarousal. Children who are relatively more affected by symptoms of re-experiencing might get more upset as a result of the intrusions, leading to emotional dysregulation and behaviour problems (Levendosky et al., 2002; Miller-Graff et al., 2016).

However, for the PTSS outcome variable, we found opposite results. Both groups of children with a disorganized attachment style had lower levels of PTSS at the start of the treatment in comparison to the two groups of children with a non-disorganized attachment style. Moreover, the level of PTSS increased throughout the treatment for the two groups of children with a disorganized attachment style. This particular finding is conflicting with previous research stating that children with a disorganized attachment style in infancy showed higher levels of PTSS at age 8.5 compared to children with a non-disorganized attachment style (MacDonald et al., 2008). However, that study’s sample differed from our sample, as it consisted of children who were intrauterine cocaine exposed. It may be speculated that PTSS are more difficult to recognize in children with a disorganized attachment style as their PTSS is more ambiguous and might be overshadowed by their behavioural problems.

Furthermore, in this exploratory study, we found several moderator effects for treatment outcomes across three of the four problem areas. In general, these results might suggest that the treatment might be more effective for children with a non-disorganized attachment style. To our knowledge, there is relatively little literature on the role of attachment style and type of core cluster PTSD symptoms in the effectiveness of treatment for CAN-related problems and therefore, our findings are difficult to interpret with certainty. Some studies found a mediating role for the change in posttraumatic cognitions behind the differing treatment outcomes between evidence-based trauma-focused treatment versus control groups (Jensen et al., 2018; Pfeiffer et al., 2017). Our study appears one of the first to research the role of attachment style and type of core cluster PTSD symptoms in the treatment of a complex population, Accordingly, we are forming the first possible hypothesizes that may clarify our findings and they can therefore be viewed as speculative.

When looking at our findings, the results suggest that a large portion of the differences in the course of symptoms throughout the treatment between children with a non-disorganized attachment style and children with a disorganized attachment style occurs in the trauma processing phase. This leads us to hypothesize that more caregiver-child sessions than the currently described number in the preparation phase of the protocol may be needed for children with a disorganized attachment style to help them shift their disorganized attachment to non-disorganized attachment patterns. Moreover, given the amount of traumatic events that characterizes our study population, it is hypothesized that a rapid and continuous course of processing of traumatic events might be overwhelming for these children. It might therefore be preferable to integrate sessions into the trauma processing phase where no actual trauma processing takes place and the focus of attention is attachment and self-regulation in order for the child to catch his or her breath. Last, a disorganized attachment style is found to be associated with dissociation (Joubert et al., 2012; MacDonald et al., 2008; Ogawa et al., 1997). Therefore, it is hypothesized that through the course of the attachment and regulation phase, children might get more awareness of their bodies and sensations. As a consequence, their symptoms may be perceived more intense and thus increase in the reports. Besides, by working with what has happened in the past, feelings of sadness, anger, and grief may arise. The treatment phase might therefore need an even longer duration. Another line of thought that crossed our mind is that it is possible that some children may need another trauma-focused approach than EMDR after the attachment and regulation phase, such as Trauma-focused Cognitive Behavioral Therapy. In the literature on adults, it is found that on individual level, patients can vary substantially between the two treatments in terms of treatment response (Deisenhofer et al., 2018). It is possible this accounts for children as well. Nevertheless, all these lines of thought are merely speculative, as this topic is scarcely researched. There are indications that a disorganized attachment style holds a stronger link with psychopathology (Borelli et al., 2010; Gazzillo et al., 2020; Zeanah et al., 2016). It might therefore be possible that a disorganized attachment style is more difficult to treat in comparison to the ambivalent or avoidant attachment styles.

As the treatment appeared more effective for the group of children with a non-disorganized attachment style and re-experiencing in terms of PTSS, it is possible that during treatment a child might cease avoiding or resisting trauma-related stimuli and therefore temporarily experience more PTSS in terms of hyperarousal and re-experiencing, leading to temporary higher reported levels of PTSS (Larsen et al., 2016). Therefore, children with re-experiencing as core cluster PTSD symptoms might benefit quicker from the trauma processing. However, this is mere speculation.

In sum, our findings contribute to the limited research available on attachment style and PTSS as possible moderators on treatment outcome. The results suggest that there are differences in the course of symptoms across the four groups our study sample consisted of, moderated by attachment style and core cluster PTSD symptoms. However, the preliminary hypotheses derived from the results of this study are in need of further research to study and elaborate these findings. Based on our results, the current treatment protocol appears to be clinically more relevant for children with a non-disorganized attachment style, and specifically for children with a non-disorganized attachment style and re-experiencing as core cluster PTSD symptoms. Despite the positive outcome for this group, it still leaves the group of children with a disorganized attachment style with unsatisfactory outcomes. This is worrisome as it is known that a disorganized attachment style may somehow be related to severe forms of psychopathology later in life (Gazzilli et al., 2020; Lecompte & Moss, 2014), although causality is not yet clear. Insufficient effectivity among interventions for attachment problems is a well-known problem, as other existing interventions that target disorganization of attachment do not significantly change or prevent disorganized attachment (Bakermans-Kranenburg et al., 2005). This highlights the long path that is still ahead in the scientific field for developing interventions for trauma – and attachment-related symptoms.

### 4.1. Limitations

We based our results on a small sample of 12 children. Therefore, our findings are preliminary and purely explorative. They need to be interpreted with caution. The limited sample size also prevented us from classifying our sample in more than the current four groups of moderator variables. Therefore, we were not able to create separate categories for children with avoidance and hyperarousal in terms of core cluster PTSD symptoms. Even though this treatment specifically focused on children with severe avoidance and dysregulation, it would be interesting to explore whether these core cluster PTSD symptoms separately account for a different symptom course. Furthermore, as this paper discusses a first experimental study of a new treatment model, the number of treatment sessions was pre-fixed. However, therapists who participated in the study informed us that when the study was finished, and the data collection was terminated, in some instances therapists continued to treat the child with EMDR as the application of EMDR with the child was possible and there were still targets to reprocess. Therefore, it might be possible we terminated the study too soon and as a consequence the full course of symptom change was missed. This might also explain the difference in results regarding PTSS between our study and the study of the original developers. In their study, some children continued treatment for up to 24 months (Wesselmann et al., 2018) and might therefore have had more time to effectively treat their trauma targets. Besides, we used the CRIES-13 to assess PTSS. The CRIES-13 is based on the DSM-IV criteria of PTSD. Therefore, we could not include DSM-5 criterion D ‘Negative alteration in cognitions and mood associated with the traumatic events. In future studies, we would make use of the Dutch version of the Child and Adolescent Trauma Screen 2 (CATS-2) which does include criterion D (Kooij & Lindauer, 2019; Sachser et al., 2017, 2022). Last, we did not include length of CAN to which the child was exposed to in the past into the variables of the participants. It is possible that this factor plays a role in explaining the different findings.

### 4.2. Recommendations for future research and clinical practice

This study highlights the importance of the continuation of the development of innovative interventions for those children for whom evidence-based interventions are insufficient. Leaving these children and their symptomatology untreated is simply not an option. We recommend future research to further investigate more personalized, innovative treatment models while simultaneously focusing on which interventions work for whom. SCED-research proved to be a useful study design to study complex populations. In recent years, SCED methodology and analyses have improved (Jamshidi et al., 2022; Kratochwill et al., 2021), which enables us to gain a richer insight in the possible course of development of different types of symptoms. As moderator analyses are also possible within SCED-research, we can be provided with a deeper understanding of why some patients respond to specific treatment modules and others do not. Contextual factors, such as familial changes or changes in living situations, should also be included as potential moderators. Especially, we call for the development of innovative and integrated treatment models for children with a disorganized attachment style. Last, as our endeavours are merely a first attempt in uncovering what works for whom in the field of CAN-related treatments, we encourage other researchers to include attachment and core cluster PTSD moderator analyses into studies on (other) trauma-focused treatments.

## 5. Conclusion

Our overall findings implicate that the integrated treatment model for trauma and attachment, that is specifically developed for a group of children with severe symptomatology who often had unsuccessful treatment attempts in the past, successfully treats children with a non-disorganized attachment style, and specifically children with a non-disorganized attachment style and re-experiencing as core cluster PTSD symptoms. However, this treatment does not capture and successfully treat every participant: there are still children who do not benefit (enough) from the treatment in its current form. This seems specifically the case for children with a disorganized attachment style. This underlines the complexity of treating children who suffer from a complicated combination of symptoms after CAN and calls for the continuous adjustment of existing treatment protocols and the development of innovative interventions for this particular group of children.

## Data Availability

Due to the nature of this research, participants of this study did not agree for their data to be shared in whole publicly, so supporting data is not available.
